# Gender, Obesity, Fat Distribution and 25-Hydroxyvitamin D

**DOI:** 10.3390/medicina59061123

**Published:** 2023-06-11

**Authors:** Maria Teresa Guagnano, Damiano D’Ardes, Pamela Di Giovanni, Ilaria Rossi, Andrea Boccatonda, Marco Bucci, Francesco Cipollone

**Affiliations:** 1 Institute of “Clinica Medica”, Department of Medicine and Aging Science, “G. D’Annunzio” University of Chieti, 66100 Chieti, Italy; guagnano@unich.it (M.T.G.); rossilaria91@gmail.com (I.R.); mbucci@unich.it (M.B.); francesco.cipollone@unich.it (F.C.); 2 Section of Hygiene, Epidemiology and Public Health, Department of Pharmacy, “G. D’Annunzio” University of Chieti, 66100 Chieti, Italy; pamela.digiovanni@unich.it; 3 Internal Medicine, Bentivoglio Hospital, AUSL Bologna, 40010 Bologna, Italy; andrea.boccatonda@gmail.com

**Keywords:** 25-hydroxyvitamin D, gender, obesity, adipose tissue

## Abstract

*Background and Objectives*: Obesity is a worldwide disease associated with systemic complications. In recent years, there has been growing interest in studying vitamin D but data related to obese subjects are still poor. AIM: The aim of this study was to evaluate the relationship between obesity degree and 25-hydroxyvitamin D [25(OH)D] levels. *Materials and Methods*: We recruited 147 Caucasian adult obese patients (BMI > 30 Kg/m^2^; 49 male; median age 53 years), and 20 overweight subjects as control group (median age 57 years), who had been referred to our Obesity Center of Chieti (Italy) between May 2020 and September 2021. *Results*: The median BMI was 38 (33–42) kg/m^2^ for obese patients and 27 (26–28) kg/m^2^ for overweight patients. 25(OH)D concentrations were lower in the obese population compared to the overweight population (19 ng/mL vs. 36 ng/mL; *p* < 0.001). Considering all obese subjects, a negative correlation was observed between 25(OH)D concentrations and obesity-related parameters (weight, BMI, waist circumference, fat mass, visceral fat, total cholesterol, LDL cholesterol) and glucose metabolism-related parameters. 25(OH)D was also negatively correlated with blood pressure. *Conclusions*: Our data confirmed the inverse relationship between obesity and blood concentration of 25(OH)D and highlighted how 25(OH)D levels decrease in the presence of glucose and lipid metabolism alterations.

## 1. Introduction

Overweight and obesity are characterized by an excessive adipose tissue accumulation that presents a risk for health. Obesity is a worldwide epidemic disease where patients show a body mass index (BMI) ≥ 30 kg/m^2^ [1]. Systemic complications, such as hypertension, dyslipidaemia, insulin resistance and diabetes mellitus, are associated with obesity [2].

Lately, there has been an important rise in attention to the study of vitamin D functions in the organism for its “calcemic” and “non-calcemic” effects [3]. Several studies have speculated a link between low vitamin D serum concentrations and different pathological conditions. Vitamin D deficiency has also been seen to be associated with autoimmune disorders, such as Hashimoto’s autoimmune thyroiditis [4]. According to recent studies, serum 25(OH)D concentrations > 75 nmol/L are associated with a low prevalence of metabolic syndrome and diabetes mellitus, with lower levels of glycated hemoglobin, insulin resistance and fasting triglycerides [5]. Even in obese children and adolescents, lower vitamin D concentrations are associated with an increase in insulin resistance [6,7]. Therefore, vitamin D plays an important role in glucose homeostasis, insulin production and obesity-related inflammation [8]. Furthermore, in an elderly population of men and women, the lowest quartiles of 25(OH)D are associated with high levels of fat mass, while insulin sensitivity is associated with the highest quartiles of vitamin D [9]. Pregnant women, obese people and individuals who are not exposed to sunlight are mainly at high risk [10].

Several studies have studied calcifediol concentrations that could be decreased in obesity: in particular, each unit of useless BMI produces a 1.15% reduction in 25(OH)D levels [11].

Fat accumulation and vitamin D deficiency contribute to produce negative effects as a result of enzymatic alterations, exceeding metabolic processes with decreased activity of alpha-hydroxylase, the principal enzyme in the transformation of calciferol in a fat-infiltrated liver, resulting in an excess of inactive forms with decreased vitamin D bioavailability [12,13,14].

Vitamin D, ingested with food or synthesized in the skin, goes into the bloodstream and undergoes two phases of hydroxylation: the first step occurs in liver forming 25(OH)D, the second one occurs in the kidneys with formation of 1,25(OH)2D. This active metabolite produces a series of classical effects (the so-called calcemic effects) and acts on phosphorus–calcium metabolism through parathormone. However, it also interacts in the tissues with vitamin D receptor (VDR), thus exerting its non-calcemic effects [15]. This active form of vitamin D acts on the kidney and it is implicated in the renin–angiotensin–aldosterone system (RAAS) regulation, coordinates immunity, affects pancreatic beta-cells and adipose cells, changes sensitivity to insulin and improves lipid profile. As a result of its pancreatic influence, insulin receptor expression is raised and insulin sensitivity is enhanced. Vitamin D controls gluconeogenesis in adipose tissue, and raises levels of HDL cholesterol and leptin [10].

However, conflicting results exist in the literature. Zarei et al. document, for example, that supplementation with vitamin D in diabetic patients does not produce significant differences in some anthropometric indices such as BMI and waist circumference [16]. Moreover, vitamin D reduces formation of insulin-like growth factor 2 [17].

Vitamin D has the role of regulating calbindin protein and works on depolarization-stimulated insulin release with redistributing intracellular calcium [18]. The previously discussed effects of vitamin D on insulin sensitivity include several mechanisms, in particular, the interaction with insulin sensitivity gene expression [19,20] and with VDR-receptor, resulting in a raised insulin receptor gene transcriptional activity that increases the overall number of insulin receptors without modifying in a substantial way their affinity [21]. 1,25 (OH)2D can also increase insulin sensitivity through peroxisome proliferator-activated receptor delta [17]. Moreover, vitamin D deficiency also leads to elevated parathormone levels, decreasing insulin sensitivity, starting lipogenesis and raising fat mass [21,22]. In addition, vitamin D acts on insulin secretion and sensitivity of tissues to insulin in obese subjects [15].

The aim of our study was to evaluate the relationship between obesity degree and blood concentrations of 25(OH)D in a sample of subjects belonging to the Obesity Center of the University Hospital of Chieti in Italy. The final objective stems from the evidence that excess weight is reaching epidemic proportions and from the need to be able to identify, already at the first medical visit, the patients at greater cardiometabolic risk in order to perform the best therapeutic actions primarily through a personalized low-calorie diet and lifestyle changes. The study also evaluated whether anthropometric measures and the indices derived from them, especially those relating to abdominal obesity, are correlated with 25(OH)D deficiency.

## 2. Material and Methods

### 2.1. Study Population

We recruited 147 Caucasian obese patients (BMI > 30) [23,24] (Male/Female 49/98) (median age 53 years) and 20 subjects with BMI ≥ 25 < 30 as control group (median age 57 years), who had been referred to the Obesity Center—University Hospital of Chieti, Italy, between May 2020 and September 2021. Inclusion criteria for obese patients were: age ≥18 years and BMI ≥ 30 Kg/m^2^.

Exclusion criteria included smoking, chronic diseases (well-controlled arterial hypertension was allowed), cancer diseases, eating disorders, serious psychiatric diseases and pregnancy or breastfeeding. Moreover, patients who also had a bariatric surgery planned or already performed were excluded. In the overweight control group, previous or recent obesity status was an exclusion criterion.

Potentially enrollable patients signed the written informed consent after an exhaustive explanation of the purpose of the study that was approved by the Ethical Committee of Chieti-Pescara (Ethics Committee Project n.7—14 may 2020).

Obese patients and control subjects underwent a baseline visit. Medical data and family history were obtained from all patients. A complete physical examination was performed with measure of anthropometric parameters.

Weight and height were calculated in patients not wearing shoes. Body weight was taken with a calibrated professional scale, and height was taken with a stadiometer to the nearest 0.1 cm. Body mass index (BMI) was calculated as the weight in kilograms divided by the square of the height in meters [24,25,26]. The circumferences, expressed in centimeters, were detected using a flexible metric tape with the subject in an upright position. In particular, the neck circumference was measured with the subject’s head in the Frankfurt plane, by applying the tape measure directly below the cricoid cartilage. The waist circumference was measured at the point immediately above the iliac crest, after a deep exhalation, with the abdominal muscles relaxed. The hip circumference was measured as the maximum circumference detectable at the level of the greater trochanter keeping the meter on a horizontal plane. The waist–hip ratio (WHR) was then calculated: a value ≥0.85 in women and ≥0.90 in men for WHR classifies subjects with distribution of fatty tissue of the android type and at greater risk of cardiovascular comorbidity compared to subjects with lower values and with distribution of fat around the hips (gynoid-type distribution) [27,28,29].

For the assessment of body composition, a bioimpedance scale (BIA SC 330 model; TANITA, Tokyo, Japan) was used, which measures the electrical resistance exerted by the body at the passage of a constant electric current at a set frequency (50 kHz, 90 μA). The values of fat mass (Fat Mass, FM), muscle mass (MM), and total body water (Total Body Water, TBW) are expressed both in Kg and in %, and they have been estimated using equations that they use a reference population (Tanita Manual). The level of visceral fat is evaluated on a numerical scale from 1 to 59. A value ≥ 13 indicates excess of visceral fat (Columbia University—New York, Istituto Tanita—Tokyo) [30].

Blood pressure was measured by the same investigator using a validated protocol. The cuff size was relative to the arm circumference and validated for obese patients [31].

In the same basal visit, obese patients were prescribed a personalized low-calorie diet. Diets gave an intake of 1400–1800 Kilocalories (Kcal)/die, and were drawn up along the current guidelines for correct composition in macronutrients [32], daily intake of cholesterol (<300 mg/die), saturated fatty acids (<10% of caloric intake per day) [33], oligosaccharides (<15% of caloric intake per day), and dietary fiber (25–30 g/die) [34]. In addition, the total protein intake was 50% from animal and 50% from vegetable proteins.

The energetic content of the meal plan was established considering the following factors: age, sex, body weight, height, physical activity grade and working activity, considering a range between a sedentary hypokinetic aspect and an aspect with forced motor involvement. Therefore, the Average Energy Requirement was computed considering the Basal Metabolism (Harris-Benedict Formula) [35] of the patients and the physical activity level. The expected caloric intake was 1200–1800 kcal per day with an average of 1450 kcal.

Obese subjects were encouraged to increase their physical activity (for example, brisk walking) for at least 2.5 h per week, to achieve at least 700 kcal/week expenditure.

Fasting blood samples of all subjects were collected at the baseline visit and frozen at −20 °C for subsequent biochemical measurements.

Blood glucose concentration was evaluated by the glucose oxidase method and serum insulin by immunochemiluminometric assays. The HbA1c was determined by automated high-performance liquid chromatography (HPLC). The oral glucose tolerance test (OGTT) was also evaluated to diagnose any impaired glucose tolerance: subjects received a 75 g glucose solution at time 0′; measurements of the plasma glucose concentration were carried out, of which the first was during fasting (time 0′ or baseline) and the other at 120 min. Insulin resistance was assessed by using the Homeostasis Model Assessment for Insulin Resistance (HOMA-IR) defined by [fasting insulin (mU/L) × fasting glucose (mmol/L)/22.5] [36,37].

Total cholesterol (T-COL), HDL cholesterol (HDL-C) and triglyceride (TG) levels were measured with enzymatic methods. LDL cholesterol (LDL-C) levels were obtained by using the Friedewald equation: LDL-C (mg/dL) = T-COL (mg/dL) − [HDL-C (mg/dL) + TG (mg/dL)/5] [38].

5P02 ARCHITECT 25-OH Vitamin D Reagent Kit (Abbott Laboratories-IL-USA) was used. The ARCHITECT 25-OH vitamin D assay is a chemiluminescent microparticle immunoassay for the quantitative determination of 25(OH)D in human serum and plasma. The control quality procedures were executed and certified by Abbott laboratories. In particular, a study of linearity was performed on guidance from NCCLS EP6-A. Sensitivity was performed based on guidance from CLSI EP17-A2. The highest observed limit of blank value was 1.6 ng/mL and the highest observed limit of detection was 2.2 ng/mL. Specificity was performed based on guidance from CLSI EP7-A2. Cross-reactivity of the ARCHITECT 25-OH Vitamin D assay with 25-OH vitamin D2 was assessed by using endogenous serum specimens.

A recommended target range of vitamin D in serum by one expert panel suggested a target range of at least 30–40 ng/mL [39].

Blood concentrations of creatinine, uric acid, vitamin D, TSH and complete blood count were measured using the analytical methods used by the Centralized Analysis Laboratory of our University Hospital.

### 2.2. Statistical Analysis

Quantitative variables were described as median and interquartile range (IQ) shown in brackets. Nonparametric statistics was used to compare the subject’s group (Mann–Whitney U-test). Pearson’s correlation coefficient was calculated to study the relationship between the variables and vitamin D concentrations. Data analysis was evaluated on GraphPad Prism 6 Software, version 6.01, 2012. The statistical significance was set at *p* = 0.05.

## 3. Results

Obese patients had a median age of 53 years (41–65); the condition of obesity had been present for 15 years (8–20). The BMI median was 38.2 (33–42) kg/m^2^ for obese patients and 26.9 (26–28) kg/m^2^ for overweight patients (Table 1).

25(OH)D was lower in the obese population compared to the overweight population [19 (13–22) ng/mL *versus* 36 (33–46) ng/mL (*p* <0.001)].

The triglycerides, insulin levels, HOMA index and systo-diastolic blood pressure were significantly elevated in obese women with respect to overweight women (*p* < 0.001).

The neck circumference was in obese subjects 41 (39–44) cm and 37 (36–39) cm in overweight patients (*p* < 0.001) (Table 1).

Waist circumference, hip circumference, fat mass, visceral fat and basal metabolism were higher in obese people than in overweight subjects (*p* < 0.001), as well as the waist to hip ratio (*p* < 0.01) (Table 1).

Instead, the HDL cholesterol was lower in obese subjects than in overweight subjects (*p* <0.05).

The correlations between 25(OH)D blood concentrations and clinical-laboratory variables in obese and overweight patients were then evaluated (Table 2).

In particular, a negative correlation was observed in obese subjects between vitamin D concentrations and obesity-related parameters (weight, BMI, waist circumference, fat mass; visceral fat; total cholesterol, LDL cholesterol) and glucose metabolism-related parameters (fasting blood glucose, blood glucose at 120 min, insulin, HOMA index).

25(OH)D was also negatively correlated with systo-diastolic blood pressure and with white blood cells (Table 2). In the overweight group, vitamin D was negatively correlated with triglycerides (*p* < 0.05).

Table 3 showed the correlation between 25(OH)D concentrations and clinical-laboratory parameters in obese android patients, both men (n. 49) and women (n. 91).

In particular, in the obese population and in both sexes, a negative correlation of 25(OH)D with BMI, waist circumference and fat mass was observed. A negative correlation was also observed, in women and men, between vitamin D and blood levels of total cholesterol, LDL-cholesterol, glycaemia at 120′ (after OGTT), insulin and HOMA Index.

There was also a negative correlation of vitamin D with triglycerides, DBP and white blood cells in android women.

In Table 4, the correlations in overweight patient are shown: there was a negative correlation of vitamin D with BMI, basal metabolic rate and SBP (*p* < 0.05) in men, while there was a negative correlation of vitamin D with triglycerides, creatinine (*p* < 0.05) and white blood cells (*p* < 0.001) in female patients.

Comparing android women (n. 91) and gynoid women (n. 7), the following was found (Table 5):

- a higher median age in android women than in gynoid women: 54 (40–63) years versus 36 (20–48) years (*p* < 0.001);
- a higher waist circumference (*p* < 0.001) in android women than in gynoid women;
- higher visceral fat level in android women than gynoid subjects (*p* < 0.05).

In the comparison between android women and men, the following was documented (Table 5):

- increase in neck circumference, greater in men than in android women: 44 (43–47) cm for men and 36 (35–42) cm for android women (*p* < 0.001);
- greater lean mass in men (66 kg) than in android women (50 kg) (*p* < 0.001) and, directly related, greater basal metabolism in men (2111 kcal) compared to android women (1705 kcal) (*p* < 0.001);
- higher level of visceral fat in men (22) than in women (11) (*p* < 0.001);
- increase in HDL cholesterol in android women (55 mg/dL) compared to men (41 mg/dL) (*p* < 0.001);
- triglycerides (*p* < 0.01), uric acid (*p* < 0.001), creatinine (*p* < 0.001) and HOMA index (*p* < 0.02) are higher in men than in android women.

## 4. Discussion and Conclusions

The purpose of this study was to demonstrate the correlation between obesity and hypovitaminosis D, a relationship already confirmed in the literature [11,12,13,14], and possibly to highlight an association between vitamin D concentrations and other individual variables.

A link between increased neck circumference and obstructive nocturnal apnea syndrome (OSAS) has been highlighted in the literature: a neck circumference > 43 cm for men and >41 cm for women, with a BMI greater than 29 kg/m^2^, is connected with an increased risk of OSAS [40]. In our study, obese patients had median neck circumference values of 41 (39–44) cm; this value puts our obese subjects at high risk of developing obstructive sleep apnea syndrome (OSAS), which has been shown to be in direct correlation with the neck circumference, due to the sagging effect of excess adipose tissue on the airways during the sleep.

The optimal values of the waist circumference are represented in men by values below 102 cm according to ATP III or below 94 cm according to the IDF and in women by values below 88 cm according to ATP III or below 80 cm according to the IDF [27]. In this study, we found values well above those recommended in both the obese and overweight populations; however, the obese are at greater risk of developing cardiovascular alterations by virtue of the significant excess of adipose tissue in the abdominal region (android conformation, WHR > 1.00).

This study confirmed what had already been found in the literature [5,11,23] regarding the correlation between blood vitamin D concentration and obesity. In addition, a recent work by Buscemi et al. confirms that obese subjects have lower concentrations of vitamin D compared to normal weight subjects [41].

It is also known that a blood concentration of vitamin D in the normal range is associated with normal lipid levels, and therefore, with a lower risk of cardiovascular morbidity and mortality [42]. Furthermore, the increase in serum 25(OH)D concentration, after weight loss induced by ketogenic diet, was also associated with an important reduction of inflammation [43].

Moreover, in our obese patients vitamin D correlated negatively with SBP and DBP. There are many vitamin D-mediated anti-atherosclerotic mechanisms such as reduction of the expression of NFkB and IL-6 in endothelial cells, increased thrombomodulin expression in macrophages and endothelial cells, reduction of the expression of tissue factor and increased endothelial production of nitric oxide [44]. CYP27B1 converts calcidiol into calcitriol, the active form of vitamin D; it was found on cells of the cardiovascular system. Its deficient expression in knock-out mice for the gene that encodes it was related to myocardial hypertrophy due to overexpression of the renin-angiotensin-aldosterone system (RAAS), hypertension, increased blood pressure thrombotic risk and progression of atherosclerosis. Precisely, vitamin D insufficiency can predispose to arterial hypertension by upregulating the RAAS system and increasing vascular resistance with subsequent vasoconstriction.

Vitamin D receptor activation also modulates myocardial contractility, probably by regulating calcium flow; low levels of calcidiol are associated with an increased overall risk of mortality from cardiovascular events. Of the many studies carried out in this regard, only the VINDICATE study reported the positive effects on left ventricle contractile function of subjects with heart failure, consequent to vitamin D supplementation [45].

Vitamin D shows a broad spectrum of cardioprotective actions, avoiding oxidative stress, thrombosis and optimizing endothelial action [46], and its deficiency could represent a further risk factor for cardiovascular comorbidities in obese patients.

All negative correlations of 25(OH)D with anthropometric measures, with some parameters of glucidic and lipid metabolism, and with cardiovascular measures such as BP, are certainly attributable to overweight/obesity status. Nevertheless, the link between excess adipose tissue and low blood concentrations of vitamin D is still not well understood. It has been hypothesized that vitamin D interacts with the expression of insulin sensitivity genes [19,20] and that hepatic 25-hydroxylase undergoes a downregulation in the presence of excess adipose tissue [47]; however, to our knowledge, there are no studies in which a significant weight loss causes an increase in vitamin D concentrations without having recommended adequate oral supplementation.

In addition, in obese patients, vitamin D deficiency has also been strongly related to systemic inflammation so much that, in the recent COVID-19 pandemic, there was a controversial debate on the possible role of vitamin D in the prevention of infection from COVID-19.

It must be underlined that our study has some limitations: (1) small number of subjects enrolled, because the observation period was still under the influence of the COVID-19 pandemic and therefore, fewer subjects underwent an obesity visit; (2) low significance in some correlations, probably due to the small number of subjects; (3) moreover, the study did not distinguish between summer and winter months; therefore, the influence of sun exposure on blood concentrations of vitamin D was not taken into account.

On the contrary, a strong point is the fact that we specifically considered in our data recording and analysis the female sex, thus aiming towards a personalized and gender-based medicine.

In conclusion, this study confirmed the negative relationship between obesity and blood concentration of vitamin D and highlighted how vitamin D blood concentrations decrease in the presence of carbohydrate and lipid alterations.

However, given the small size of this study, further studies are needed to add data and strengthen our observations.

## Figures and Tables

**Table 1:** Clinical and laboratory parameters.

	Obeses Subjects	Overweight Subjects	*p*-Value
Number	147	20	
Male/Female	49/98	10/10	-
Age (years)	53 (41–65)	57 (47–70)	NS ^i^
Duration of Obesity (years)	15 (8–20)	-	-
Weight (Kg)	99 (87–116)	72 (66–78)	<0.001
Height (cm)	163 (156–171)	164 (157–169)	NS
BMI (Kg/m^2^) ^a^	38 (34–42)	27 (26–28)	<0.001
Neck Circumference (cm)	41 (39–44)	37 (36–39)	<0.001
Waist Circumference (cm)	121 (113–131)	99 (93–101)	<0.001
Hip Circumference (cm)	121 (113–130)	100 (97–106)	<0.001
WHR ^b^	1 (0.9–1.1)	1 (0.9–1.0)	0.001
Fat Mass (Kg)	42 (35–53)	21 (17–25)	<0.001
Free Fat Mass (Kg)	54 (47–61)	47 (40–59)	0.019
Visceral Fat (level) ^c^	15 (12–21)	10 (7–12)	<0.001
Basal Metabolism (Kcal)	1745 (1532–2001)	1454 (1273–1785)	<0.001
Total Cholesterol (mg/dL)	201 (171–219)	191 (161–206)	NS
HDL Cholesterol (mg/dL)	47 (40–55)	52 (46–65)	0.026
LDL Cholesterol (mg/dL)	124 (102–142)	118 (84–129)	NS
Triglycerides (mg/dL)	124 (96–166)	85 (61–122)	0.001
Uric Acid (mg/dL)	6 (5–7)	5 (4–6)	0.022
Creatinine (mg/dL)	1 (0.7–0.9)	1 (0.7–0.9)	NS
FBG (mg/dL) ^d^	96 (90–106)	97 (89–100)	NS
OGTT 120′ (mg/dL) ^e^	119 (104–148)	120 (99–135)	NS
Insulin (µU/mL)	16 (10–22)	8 (5–14)	<0.001
HOMA INDEX	4 (2–6)	2 (1–3)	<0.001
TSH (µU/mL) ^f^	2 (1–3)	2 (1–2)	NS
25(OH)D (ng/mL)	19 (14–23)	36 (33–47)	<0.001
SBP (mmHg) ^g^	140 (120–145)	117 (110–124)	<0.001
DBP (mmHg) ^h^	80 (80–90)	70 (70–80)	<0.001

Median and interquartile range—IQ. ^a^ BMI: Body Mass Index; ^b^ WHR: Waist–Hip Ratio; ^c^ Visceral Fat: range 1–59; ^d^ FBG: Fasting Blood Glucose; ^e^ OGTT: Oral Glucose Tolerance Test; ^f^ TSH: Thyroid Stimulating Hormone; ^g^ SBP: Systolic Blood Pressure; ^h^ DBP: Diastolic Blood Pressure; ^i^ NS: not significant. *p*-value derived from Mann–Whitney test. Statistical significance *p* = 0.05.

**Table 2:** Correlation between 25(OH)D concentrations and clinical-laboratory parameters in obese and overweight subjects.

	Obeses Subjects (*n* = 147)		Overweight Subjects (*n* = 20)	
	rho	*p*-Value	rho	*p*-Value
Weight (kg)	−0.20	<0.05	−0.23	NS ^i^
Height (cm)	−0.01	NS	−0.17	NS
BMI (kg/m^2^) ^a^	−0.22	<0.001	−0.16	NS
Neck Circumference (cm)	−0.10	NS	−0.25	NS
Waist Circumference (cm)	−0.23	0.006	0.04	NS
Hip Circumference (cm)	−0.15	NS	0.18	NS
WHR ^b^	−0.13	NS	−0.10	NS
Fat Mass (kg)	−0.24	<0.001	0.23	NS
Free Fat Mass (kg)	−0.07	NS	−0.40	NS
Visceral Fat (level) ^c^	−0.21	<0.05	−0.01	NS
Basal Metabolism (kcal)	−0.12	NS	−0.41	NS
Total Cholesterol(mg/dL)	−0.16	<0.05	−0.04	NS
HDL-Cholesterol(mg/dL)	0.15	NS	0.18	NS
LDL-Cholesterol (mg/dL)	−0.17	<0.05	−0.01	NS
Triglycerides (mg/dL)	−0.15	NS	−0.46	<0.05
Uric Acid (mg/dL)	−0.05	NS	−0.32	NS
Creatinine (mg/dL)	0.01	NS	−0.25	NS
FBG (mg/dL) ^d^	−0.20	<0.05	−0.24	NS
OGTT-120′(mg/dL) ^e^	−0.32	<0.001	−0.03	NS
Insulin (µU/mL)	−0.32	<0.001	−0.22	NS
HOMA INDEX	−0.36	<0.001	−0.24	NS
TSH (µU/mL) ^f^	0.01	NS	0.17	NS
SBP (mmHg) ^g^	−0.21	<0.05	0.04	NS
DBP (mmHg) ^h^	−0.26	<0.001	−0.05	NS
White blood cells (10^3^/L)	−0.24	<0.001	−0.44	NS

^a^ BMI: Body Mass Index; ^b^ WHR: Waist–Hip Ratio; ^c^ Visceral Fat: range 1–59; ^d^ FBG: Fasting Blood Glucose; ^e^ OGTT: Oral Glucose Tolerance Test; ^f^ TSH: Thyroid Stimulating Hormone; ^g^ SBP: Systolic Blood Pressure; ^h^ DBP: Diastolic Blood Pressure; ^i^ NS: Not Significant. Pearson’s correlation coefficients. Statistical significance *p* = 0.05.

**Table 3:** Correlation between 25(OH)D concentrations and clinical-laboratory parameters in android obese women and obese men.

	Android Obese Women (*n* = 91)		Obese Men (*n* = 49)	
	rho	*p*-Value	rho	*p*-Value
Weight (kg)	−0.15	NS	−0.28	NS ^i^
Height (cm)	−0.01	NS	−0.12	NS
BMI (kg/m^2^) ^a^	−0.26	<0.05	−0.34	<0.05
Neck Circumference (cm)	−0.05	NS	−0.16	NS
Waist Circumference (cm)	−0.28	<0.05	−0.29	<0.05
Hip Circumference (cm)	−0.09	NS	−0.30	<0.05
WHR ^b^	−0.14	NS	−0.03	NS
Fat Mass (kg)	−0.27	<0.001	−0.36	<0.05
Free Fat Mass (kg)	−0.05	NS	−0.04	NS
Visceral Fat (level) ^c^	−0.11	NS	−0.36	<0.05
Basal Metabolism (kcal)	−0.12	NS	−0.12	NS
Total Cholesterol (mg/dL)	−0.28	<0.05	−0.32	<0.05
HDL-Cholesterol (mg/dL)	0.16	NS	0.11	NS
LDL-Cholesterol (mg/dL)	−0.30	<0.05	−0.30	<0.05
Triglycerides (mg/dL)	−0.20	<0.05	−0.20	NS
Uric Acid (mg/dL)	0.01	NS	−0.07	NS
Creatinine (mg/dL)	−0.01	NS	0.13	NS
FBG (mg/dL) ^d^	−0.23	<0.05	−0.20	NS
OGTT-120′ (mg/dL) ^e^	−0.27	<0.001	−0.37	<0.001
Insulin (µU/mL)	−0.30	<0.001	−0.38	<0.001
HOMA INDEX	−0.34	<0.001	−0.42	<0.001
TSH (µU/mL) ^f^	−0.01	NS	0.03	NS
SBP (mmHg) ^g^	−0.19	NS	−0.26	NS
DBP (mmHg) ^h^	−0.26	<0.05	−0.27	NS
White blood cells (10^3^/L)	−0.24	<0.05	−0.24	NS

^a^ BMI: Body Mass Index; ^b^ WHR: Waist–Hip Ratio; ^c^ Visceral Fat: range 1–59; ^d^ FBG: Fasting Blood Glucose; ^e^ OGTT: Oral Glucose Tolerance Test; ^f^ TSH: Thyroid Stimulating Hormone; ^g^ SBP: Systolic Blood Pressure; ^h^ DBP: Diastolic Blood Pressure; ^i^ NS: Not Significant. Pearson’s correlation coefficients. Statistical significance *p* = 0.05.

**Table 4:** Correlation between 25(OH)D concentrations and clinical-laboratory parameters in overweight women and men.

	Overweight Women (*n* = 10)		Overweight Men (*n* = 10)	
	rho	*p*-Value	rho	*p*-Value
Weight (kg)	0.12	NS	−0.40	NS ^i^
Height (cm)	0.19	NS	−0.20	NS
BMI (kg/m^2^) ^a^	−0.05	NS	−0.55	<0.05
Neck Circumference (cm)	−0.01	NS	−0.26	NS
Waist Circumference (cm)	0.22	NS	0.16	NS
Hip Circumference (cm)	0.31	NS	−0.30	NS
WHR ^b^	0.07	NS	0.38	NS
Fat Mass (kg)	0.16	NS	0.01	NS
Free Fat Mass (kg)	−0.19	NS	−0.56	NS
Visceral Fat (level) ^c^	0.07	NS	0.62	NS
Basal Metabolism (kcal)	−0.16	NS	−0.62	<0.05
Total Cholesterol (mg/dl)	0.05	NS	−0.36	NS
HDL-Cholesterol (mg/dl)	0.26	NS	−0.21	NS
LDL-Cholesterol (mg/dl)	0.06	NS	−0.24	NS
Triglycerides (mg/dl)	−0.65	<0.05	−0.49	NS
Uric Acid (mg/dL)	−0.31	NS	−0.08	NS
Creatinine (mg/dL)	−0.63	<0.05	0.58	NS
FBG (mg/dl) ^d^	−0.37	NS	−0.14	NS
OGTT-120′ (mg/dl) ^e^	−0.21	NS	0.16	NS
Insulin (µU/mL)	−0.43	NS	−0.15	NS
HOMA INDEX	−0.45	NS	−0.14	NS
TSH (µU/mL) ^f^	0.22	NS	−0.08	NS
SBP (mmHg) ^g^	−0.07	NS	0.67	<0.05
DBP (mmHg) ^h^	−0.13	NS	−0.06	NS
White blood cells (10^3^/L)	−0.77	<0.001	−0.01	NS

^a^ BMI: Body Mass Index; ^b^ WHR: Waist–Hip Ratio; ^c^ Visceral Fat: range 1–59; ^d^ FBG: Fasting Blood Glucose; ^e^ OGTT: Oral Glucose Tolerance Test; ^f^ TSH: Thyroid Stimulating Hormone; ^g^ SBP: Systolic Blood Pressure; ^h^ DBP: Diastolic Blood Pressure; ^i^ NS: Not Significant. Pearson’s correlation coefficients. Statistical significance *p* = 0.05.

**Table 5:** Clinical and laboratory parameters in women (android–gynoid) and men.

	Android Women	Gynoid Women	*p*-Value ^(1)^	Men	*p*-Value ^(2)^
Number	91	7	-	49	
Age (years)	54 (40–65)	36 (20–48)	0.006	53 (44.5–67)	NS
Duration of Obesity (years)	12 (8–20)	10 (6–20)	NS	15 (8–30)	NS
Weight (kg)	94.2 (82.8–110)	98.3 (89.5–110.8)	NS	113 (96.9–132.6)	<0.001
Height (cm)	158 (152–162)	163 (158–166)	NS	172 (166–177)	<0.001
BMI (kg/m^2^)	38.3 (34.2–42.1)	36.1 (32.4–42.7)	NS	38.2 (33.4–43.3)	NS
Neck Circumference (cm)	39 (38–42)	36 (35–42)	NS	44 (43–47)	<0.001
Waist Circumference (cm)	121 (113–128)	103 (96–110)	<0.001	127 (116–138)	0.014
Hip Circumference (cm)	121 (116–130)	130 (128–137)	0.028	116 (109.5–127.5)	0.048
WHR	0.9 (0.9–1.0)	0.8 (0.7–0.8)	<0.001	1.04 (1.0–1.1)	<0.001
Fat Mass (kg)	42.7 (35.6–53.1)	42.4 (36.7–54.7)	NS	39.3 (32.4–53.2)	NS
Free Fat Mass (kg)	49.4 (44.1–53.9)	50.1 (47.2–55.1)	NS	66.1 (58.8–75.8)	<0.001
Visceral Fat (level)	13 (11–16)	11 (8–12)	0.032	22 (18–26.5)	<0.001
Basal Metabolism (kcal)	1615 (1415–1781)	1705 (1527–1845)	NS	2111 (1864–2459.5)	<0.001
Total Cholesterol (mg/dL)	201 (179–223)	186 (158–202)	NS	202 (168.5–219.5)	NS
HDL-Cholesterol (mg/dL)	51 (44–57)	55 (43–65)	NS	41 (36.5–46)	<0.001
LDL-Cholesterol (mg/dL)	125.8 (101.8–141.6)	112.4 (72.6–129.2)	NS	123.4 (103.1–147.3)	NS
Triglycerides (mg/dL)	118 (87–146)	103 (79–128)	NS	146 (116–189.5)	0.001
Uric Acid (mg/dL)	5.3 (4.6–6.1)	4.5 (3.2–5.7)	NS	6.4 (5.6–7.4)	<0.001
Creatinine (mg/dL)	0.7 (0.6–0.8)	0.7 (0.7–0.8)	NS	0.9 (0.8–1)	<0.001
FBG (mg/dL)	94 (90–105)	91 (88–101)	NS	100 (93–115.5)	0.010
OGTT-120′ (mg/dL)	119 (110–143)	113 (66–127)	NS	117 (93.5–150)	NS
Insulin (µU/mL)	15 (10–20)	8 (7–13)	NS	17 (11.1–26)	NS
HOMA INDEX	3.6 (2.3–5.2)	1.9 (1.4–3.3)	NS	4.4 (2.8–6.4)	0.010
TSH (µU/mL)	2.1 (1.4–2.9)	1.6 (1.3–4.2)	NS	2.1 (1.1–2.7)	NS
Vitamin D (ng/mL)	19.7 (13.8–22.7)	19 (16–23.8)	NS	18.5 (12.3–22.8)	NS
SBP (mmHg)	130 (120–145)	130 (130–140)	NS	140 (127.5–150)	NS
DBP (mmHg)	80 (80–90)	80 (80–85)	NS	80 (75–92.5)	NS

BMI: Body Mass Index; WHR: Waist–Hip Ratio; Visceral Fat: range 1–59; FBG: Fasting Blood Glucose; OGTT: Oral Glucose Tolerance Test; TSH: Thyroid Stimulating Hormone; SBP: Systolic Blood Pressure; DBP: Diastolic Blood Pressure; NS: Not Significant; (1) *p*-value derived from Mann–Whitney test between android women and ginoid women. (2) *p*-value derived from Mann–Whitney test between android women and men.

## Data Availability

The data presented in this study are available on request from the corresponding author.
